# Thermoelectric Enhancements in PbTe Alloys Due to Dislocation‐Induced Strains and Converged Bands

**DOI:** 10.1002/advs.201902628

**Published:** 2020-05-15

**Authors:** Yixuan Wu, Pengfei Nan, Zhiwei Chen, Zezhu Zeng, Ruiheng Liu, Hongliang Dong, Li Xie, Youwei Xiao, Zhiqiang Chen, Hongkai Gu, Wen Li, Yue Chen, Binghui Ge, Yanzhong Pei

**Affiliations:** ^1^ Interdisciplinary Materials Research Center School of Materials Science and Engineering Tongji University 4800 Caoan Rd. Shanghai 201804 China; ^2^ Institute of Physical Science and Information Technology Anhui University Hefei 230601 China; ^3^ Department of Mechanical Engineering The University of Hong Kong Pokfulam Road Hong Kong SAR China; ^4^ State Key Laboratory of High Performance Ceramics and Superfine Microstructure Shanghai Institute of Ceramics CAS Shanghai 200050 China; ^5^ Center for High Pressure Science & Technology Advanced Research Shanghai 201203 China; ^6^ State Key Laboratory of Superhard Materials Jilin University Changchun 130012 China

**Keywords:** band convergence, dislocations, lattice strain, PbTe, thermoelectronics

## Abstract

In‐grain dislocation‐induced lattice strain fluctuations are recently revealed as an effective avenue for minimizing the lattice thermal conductivity. This effect could be integratable with electronic enhancements such as by band convergence, for a great advancement in thermoelectric performance. This motivates the current work to focus on the thermoelectric enhancements of p‐type PbTe alloys, where monotelluride‐alloying and Na‐doping are used for a simultaneous manipulation on both dislocation and band structures. As confirmed by synchrotron X‐ray diffractions and Raman measurements, the resultant dense in‐grain dislocations induce lattice strain fluctuations for broadening the phonon dispersion, leading to an exceptionally low lattice thermal conductivity of ≈0. 4 W m‐K^−1^. Band structure calculations reveal the convergence of valence bands due to monotelluride‐alloying. Eventually, the integration of both electronic and thermal improvements lead to a realization of an extraordinary figure of merit *zT* of ≈2.5 in Na_0.03_Eu_0.03_Cd_0.03_Pb_0.91_Te alloy at 850 K.

## Graphical abstract

Simultaneous manipulation of in‐grain dislocations for lattice thermal conductivity reduction and band convergence for electronic enhancement leads to a realization of an extraordinary thermoelectric figure of merit in p‐PbTe alloys.

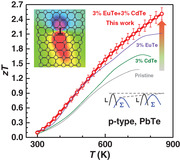

## 1 Introduction

Due to the capability of a direct conversion between heat and electricity without any emissions or moving parts, thermoelectric energy conversion for both power generation and refrigeration applications has attracted increasing attentions in this century.^[^ ^1^ ^]^ Because a thermoelectric device consists of both n‐ and p‐type semiconductors, its conversion efficiency depends straightforward on the thermoelectric performance of constituent materials, which is characterized by the dimensionless figure of merit, *zT* = *S* ^2^σ*T*/(*κ_E_* + *κ_L_*). In the equation, *S*, σ, κ_E_, and κ_L_ are the Seebeck coefficient, electrical conductivity, absolute temperature, electronic and lattice components of thermal conductivity, respectively.^[^ ^2^ ^]^

Due to the strong correlation among *S*, σ, and *κ_E_*, an effective enhancement in *zT* through an individual improvement of these parameters is challenging. This leads existing efforts to largely focus on a suppression of lattice thermal conductivity (*κ_L_*) for advancing thermoelectric materials, since *κ_L_* is the only independent parameter determining *zT*. This has been proven to be particularly effective in exploring new materials with an intrinsically low *κ_L_*,^[^ ^3^ ^]^ due to a strong inherent lattice anharmonicity,^[^ ^4^ ^]^ a slow phonon group velocity^[^ ^5^ ^]^ stemming from weakly bonded heavy atoms^[^ ^6^ ^]^ and a low fraction of acoustic phonons^[^ ^7^ ^]^ resulting from a complex crystal structure.^[^ ^8^ ^]^ Alternatively, strengthening the scattering of phonons,^[^ ^9^ ^]^ such as by introducing various dimensional defects (0D point defects,^[^ ^10^ ^]^ 1D dislocations,^[^ ^11^ ^]^ and 2D interfaces of nanostructures,^[^ ^12^ ^]^ has also been found to be successful.

Recently, the broadening of phonon dispersion induced by defects is revealed as a fundamental measure of phonon scattering for *κ_L_*‐reduction.^[^ ^10^, ^13^ ^]^ For the simple case of single atom chain with *k_c_* as the cut‐off wave vector, *m* as the atomic mass and *f* as the atomic interaction force constant, the phonon dispersion (frequency ω versus wave vector *k*) is *ω* = 2(*f*/*m*)^0.5^sin(*πk*/2*k_c_*). Ground‐state phonon dispersion at 0 K is a curve without a width. However due to the intrinsic existence of lattice anharmonicity in real crystals, atomic vibrations at finite temperatures lead to fluctuations in force constant thus fluctuations in phonon frequency near its ground state. This leads to a widening of the phonon dispersion curve. Once the crystal includes imperfections,^[^ ^2^, ^14^ ^]^ it could induce additional extrinsic fluctuations in atomic mass and/or in interaction force constant for a further broadening of the dispersion curve.^[^ ^10^, ^13^ ^]^ Such a dispersion broadening accelerates phonons to relax back to their equilibrium states (shortening in phonon lifetime or strengthening in phonon scattering), since available frequencies become much more diversified.^[^ ^15^ ^]^

Therefore, effective sources for maximizing phonon scattering are defects that enable large fluctuations in atomic mass and/or force constant.^[^ ^13a^ ^]^ Thermoelectric semiconductors usually require a carrier concentration of ≈10^20^ cm^−3^,^[^ ^16^ ^]^ which corresponds to a concentration of <1% charged dopants. As a result, higher concentration of point defects involved in thermoelectrics are mostly isovalent ones, leading the force constant fluctuations to be mostly enabled by strain (atomic distance) fluctuations. Surely point defects could enable very large mass fluctuations but the strain term is usually released due to the overall lattice relaxation (i.e., either expansion or shrinkage via the Vegard's law).

In many cases, a very high point defect concentration in thermoelectrics is not welcome due to their detrimental effects on electronic properties (such as band structure^[^ ^17^ ^]^ and mobility^[^ ^18^ ^]^) or is disabled due to the limited solubility. This indicates the importance of large strain fluctuations by other types of defects including 1D dislocations and 2D interfaces.^[^ ^19^ ^]^ The former induce large strain fluctuations^[^ ^10^, ^13^ ^]^ and the later can be considered as aligned arrays of the former with a rotation angle. Without varying much in composition, dislocations are therefore found to be particularly effective for advancing thermoelectrics,^[^ ^11^, ^20^ ^]^ largely through the resultant strain fluctuations for an effective phonon scattering (i.e., broadening in phonon dispersion^[^ ^13a^ ^]^).

From the electronic aspect, band engineering^[^ ^17^, ^21^ ^]^ approaches including convergence and nestification^[^ ^22^ ^]^ are known to be particularly effective for realizing a high band degeneracy (*Nv*) for an enhanced conductivity without explicitly decreasing Seebeck coefficient. This has led to great improvements in many thermoelectric materials such as PbTe,^[^ ^23^ ^]^ SnTe,^[^ ^24^ ^]^ GeTe,^[^ ^25^ ^]^ PbSe,^[^ ^11b^ ^]^ Mg_2_Si,^[^ ^26^ ^]^ Mg_3_Sb_2_,^[^ ^27^ ^]^ CoSb_3_,^[^ ^28^ ^]^ Bi_2_Te_3_,^[^ ^29^ ^]^ and half‐Heuslers.^[^ ^30^ ^]^

PbTe‐based materials have the capability for demonstrating the concepts including both band and microstructure engineering approaches. Recent literatures achieving an efficiency as high as 8–9% on single‐stage 2 × 4 pair PbTe module could be a good demonstration of the high performance of PbTe materials with peak *zT*s of 1.8–2.0 for p‐type and 1.2–1.4 for n‐type under a temperature difference of 570–590 K.^[^ ^31^ ^]^ In case of p‐type PbTe, alloying with monotellurides such as MnTe,^[^ ^32^ ^]^ MgTe,^[^ ^33^ ^]^ CdTe,^[^ ^34^ ^]^ EuTe,^[^ ^35^ ^]^ YbTe,^[^ ^36^ ^]^ and SrTe^[^ ^37^ ^]^ is found to be successful for reducing the energy offset between the *L* and Σ valence bands thus for increasing the electronic performance.

Among these effective alloying agents for converging the valence bands of PbTe, CdTe is very interesting because of its strong temperature dependent solubility in PbTe according to literature phase diagrams.^[^ ^38^ ^]^ This suggests an existence of transition of dominant defect types from precipitates to substitutions as temperature rises,^[^ ^39^ ^]^ similar behavior of which is also found in the SnTe‐CdSe^[^ ^24a^ ^]^ system. In addition, EuTe is known to be unique as well, since it enables the formation of dense in‐grain lattice dislocations in p‐type PbTe that lead to an extremely efficient reduction in *κ_L_*.^[^ ^35^ ^]^

It is therefore motivated in this work that an involvement of both EuTe‐ and CdTe‐alloying not only optimizes the valence band structure of PbTe solid solutions at high temperatures, but also regulates the defect structures involving both dense in‐grain dislocations and precipitate‐induced interfaces at low temperatures for understanding their effects on lattice strains for *κ_L_* ‐reduction.

## 2 Results and Discussions

**Figure 1:**
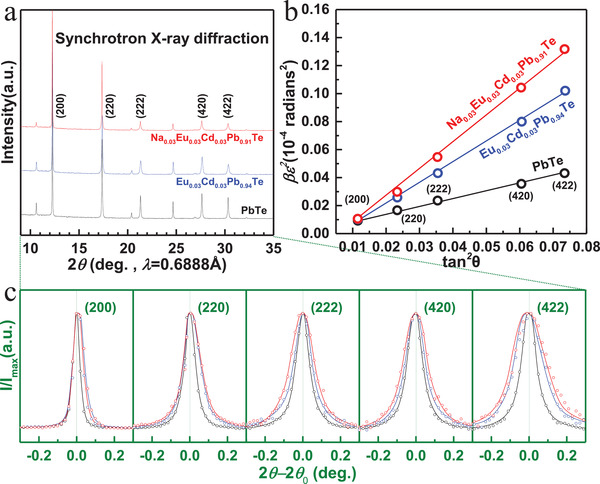
a) Room‐temperature synchrotron X‐ray diffraction patterns and b) the corresponding lattice strain analyses with c) detailed broadening in diffraction peaks for PbTe, Cd_0.03_Eu_0.03_Pb_0.94_Te and Na_0.03_Cd_0.03_Eu_0.03_Pb_0.91_Te.

The details on materials synthesis, characterization, transport‐property measurements, and band structure calculations are given in the Supporting Information. The powder X‐ray diffraction (XRD) patterns by both a synchrotron radiation facility and a laboratory one are shown in **Figure** **1**; Figure S1, Supporting Information, respectively. From the XRD patterns, all the diffraction peaks for Na_0.02_Eu_0.03_Cd*_x_*Pb_0.95−_ *_x_*Te and Na*_y_*Eu_0.03_Cd_0.03_Pb_0.94−_ *_y_*Te can be well indexed to the rock salt structure.

Lattice thermal conductivity (*κ_L_*), is fundamentally determined by the phonon dispersion, of which the slope at a given wave vector determines the corresponding phonon group velocity and the degree of broadening governs the scattering rate (lifetime).^[^ ^13a^ ^]^ Apart from the inherent lattice anharmonicity that enables an intrinsic broadening in phonon dispersion, various defects additionally induce an extrinsic broadening due to the introduced mass and/or strain fluctuations for a further *κ_L_*‐reduction.^[^ ^10^, ^40^ ^]^ Without a variation in the matrix composition, p‐type PbTe alloys with fixed content of 3% CdTe + 3% EuTe are focused on in this work, not only because of its high thermoelectric performance (Figure S2, Supporting Information) but also the minimal effect involved on *κ_L_*‐reduction due to mass fluctuations.

In order to macroscopically estimate the lattice strain fluctuations induced by defects and the contribution on *κ_L_*, both X‐ray diffraction and Raman spectroscopy techniques are used. Once lattice strain fluctuations exit, a broadening and a reduced intensity of XRD diffraction peaks can be observed.^[^ ^41^ ^]^ Since point defects are usually uniformly distributed and lead to a lattice expansion/contraction (known as the Vegard's law) for releasing the strain energy, the most observable changes in XRD patterns are the corresponding shifts in Bragg diffraction angles.

Both dislocations and interfaces could induce strain fluctuations for widening the X‐ray diffraction peaks, but the degree of broadening due to the former reason largely depends on the diffraction order while that due to the latter does not.^[^ ^41^ ^]^ This enables a distinguishability of lattice strain fluctuations due to either dislocations or interfaces, respectively, through the slope and intercept of peak broadening versus diffraction order^[^ ^41^ ^]^ as shown in Figure 1b. In addition, the grain sizes for the materials here are comparable and coarse enough according to the SEM observations (Figure S3, Supporting Information), validating a direct comparison on “full width at half maximum” (FWHM) by dislocation among different samples. Figure 1c shows the detailed peak broadening versus the diffraction indexes, where β is the FWHM of the intense diffraction peaks and θ is the Bragg angle (more details in the Supporting Information). The larger in slope of the lines in Figure 1b corresponds to the larger lattice stain fluctuations induced by a higher concentration of dislocations.^[^ ^41^ ^]^ More details on lattice strain estimation by XRD is given in the Supporting Information, and the net lattice strain due to doping is obtained by subtracting that of pristine PbTe. Mechanical properties obtained by averaging five measurements indicate that CdTe‐alloying improves the hardness by about 30% and the formation of dense in‐grain dislocations here weakly affects the strength (Figure S4, Supporting Information).

**Figure 2:**
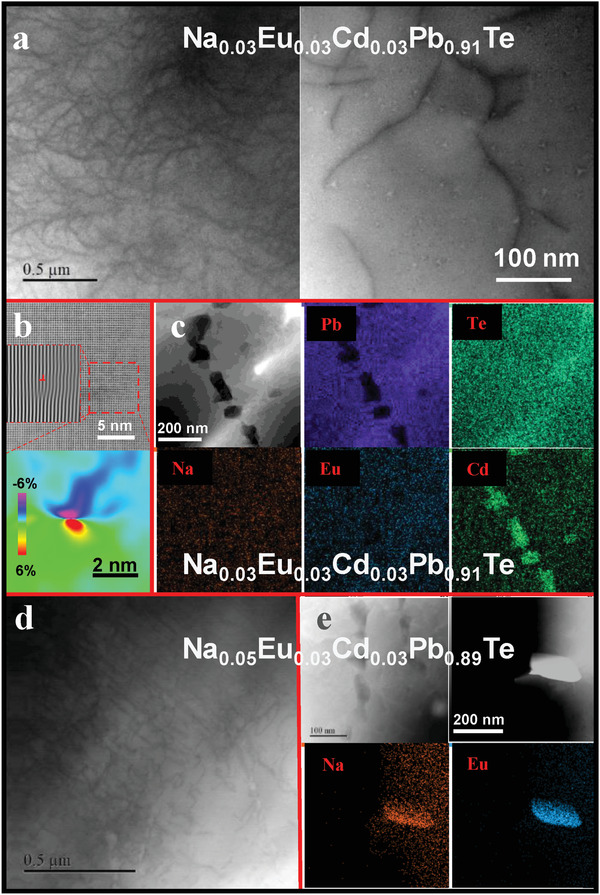
a) STEM images for Na_0.03_Eu_0.03_Cd_0.03_Pb_0.91_Te showing the coexistence of both dense dislocations and b) nano‐precipitates and the corresponding strain mappings due to a typical dislocation and c) EDS mappings for the CdTe precipitates; d) STEM images for Na_0.05_Eu_0.03_Cd_0.03_Pb_0.89_Te showing dislocations and e) EDS mappings for the Eu‐ and Na‐rich precipitates.

Scanning transmission electron microscopy (STEM) is employed to reveal the origin of lattice strains. Both dense in‐grain dislocations and nano‐precipitates (**Figure** **2**a) are observed in the high‐*zT* composition Na_0.03_Eu_0.03_Cd_0.03_Pb_0.91_Te. Based on a geometric phase analysis (GPA), which is a semiquantitative lattice image‐processing approach taking into account both real‐ and reciprocal‐space information, high‐magnification STEM images enable a local estimation of the very large lattice strain fluctuations induced by dislocations through the GPA analysis (Figure 2b). The strain fluctuations due to a 2D interface are presumably much less, since it can be approximated as aligned dislocation arrays leading the net lattice strains to be those between grains with a rotation angle.^[^ ^10b^ ^]^

It should be noted that a direct observation of dislocation formation is challenging, it is usually believed that either a vacancy/interstitial clustering^[^ ^42^ ^]^ or a plastic deformation could favor the formation. In this work, since brittle PbTe thermoelectrics disable a plastic deformation, the observed dense in‐grain dislocations are believed to be due to solid‐state defect processes. Na‐doping here would lead to an increase in the formation of oppositely charged anion vacancies due to charge compensation, thus promoting the nucleation of dislocations. These charged defects also have strong electrostatic interactions with the charged dislocation segments that have climbed a half unit cell,^[^ ^43^ ^]^ which could help stabilize the dislocations.^[^ ^35^, ^43^, ^44^ ^]^

**Figure 3:**
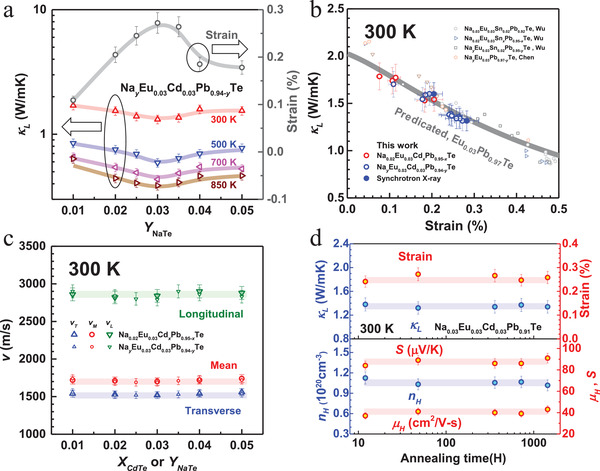
a) Composition‐dependent lattice thermal conductivity and lattice strains for Na*_y_*Eu_0.03_Cd_0.03_Pb_0.94−_ *_y_*Te; b) room‐temperature lattice strain dependent *κ_L_* with a comparison to model prediction for Eu_0.03_Pb_0.97_Te and literature results for PbTe alloys;^[^ ^13^, ^35^ ^]^ c) composition‐dependent sound velocity for Na_0.02_Eu_0.03_Cd*_x_*Pb_0.95−_ *_x_*Te and Na*_y_*Eu_0.03_Cd_0.03_Pb_0.94−_ *_y_*Te; d) annealing time (at 900 K) dependent lattice strain, lattice thermal conductivity (*κ_L_*), carrier concentration (*n_H_*), Hall mobility (*μ_H_*), and Seebeck coefficient (*S*) for the high performance composition Na_0.03_Eu_0.03_Cd_0.03_Pb_0.91_Te at room temperature.

Nano‐precipitates in Na_0.03_Eu_0.03_Cd_0.03_Pb_0.91_Te are CdTe according to the energy dispersion spectrum (EDS) analyses as shown in Figure 2c, which is similar with the cases in literature works on PbTe^[^ ^34^ ^]^ and SnTe^[^ ^24a^ ^]^ with CdTe‐alloying. With a further increase in Na‐doping concentration, a likely reduced dislocation density (Figure 2d) and additional precipitates rich in Eu and Na (Figure 2e) are observed in Na_0.05_Eu_0.03_Cd_0.03_Pb_0.89_Te, both of which are observable in Na‐doped PbTe‐EuTe alloys.^[^ ^35^ ^]^ Such a transition of defect structures from 1D dislocations to 2D interfaces^[^ ^35^ ^]^ enables an evaluation of their different effects on lattice strain fluctuations thus the reduction in lattice thermal conductivity (**Figure** **3**a). It is seen that the lattice strain maximizes at a Na concentration of 3%, indicating that nano‐precipitates do not introduce a significant contribution to lattice strain fluctuations.

Being of great importance, the increase in lattice strain is found to dominate the decrease in *κ_L_* (Figure 3a,b). Here, *κ_L_* is estimated through subtracting the electronic component according to the Wiedemann–Franz law (*κ_E_* = *LT*/ρ) from total thermal conductivity, where *L* is the Lorenz factor determined based on a single parabolic band model (SPB) with acoustic scattering (Figure S5, Supporting Information). With a nearly unchanged sound velocity due to alloying and doping (Figure 3c), the increase in lattice strain fluctuations is found to indeed take the critical responsibility for the decrease in *κ_L_*, which agrees well with the literature results^[^ ^13a^ ^]^ and can further be well predicted by the *κ_L_* ‐modeling without any fitting parameters (Figure 3b). The spectral lattice thermal conductivity taking into account the effects of mass and strain fluctuations is shown in Figure S6, Supporting Information, for Na_0.03_Eu_0.03_Cd_0.03_Pb_0.91_Te at 300, 500, 700, and 850 K. It can be seen that the strain fluctuations take the main responsibility for the reduction in spectral lattice thermal conductivity, particularly in the low‐frequency range. Details on the *κ_L_*‐modeling are given in Table S1, Supporting Information. Note here *κ_L_* as low as 0.4 W m‐K^−1^ at high temperatures is achieved, which is one of the lowest ever reported for p‐PbTe thermoelectrics. In addition, since the bonding energy in compounds is generally much larger as compared to that in metals, the equilibrium of dislocations is expected to take much longer in compound semiconductors including PbTe thermoelectrics (at an order of 10 h). Similar to that in previous works,^[^ ^35^ ^]^ dislocations in this work are found to be stable and equilibrized, since a long‐term annealing up to 2 months at a high temperature (900 K) does not reduce the lattice strains or other transport properties as detailed in Figure 3d.

**Figure 4:**
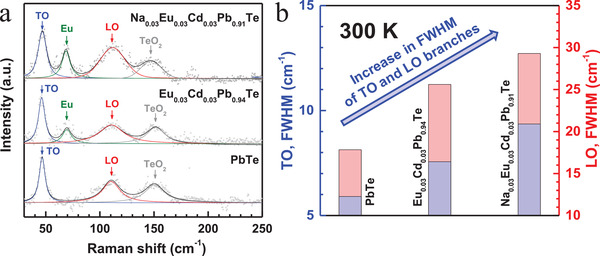
a) Room temperature Raman spectrum with Lorentzian deconvolutions and b) the corresponding Raman peak broadening versus lattice strains for pristine PbTe, Eu_0.03_Cd_0.03_Pb_0.94_Te, and Na_0.03_Eu_0.03_Cd_0.03_Pb_0.91_Te.

Lattice strains result in fluctuations in atomic distance thus interaction forces, which would further cause the fluctuations in phonon frequencies (the broadening of phonon dispersion) thus the reduction of phonon lifetime. Therefore, the Raman peak broadening induced by lattice strains (**Figure** **4**) can be a direct evidence for the phonon dispersion broadening, which reduces the phonon relaxation time and the lattice thermal conductivity. This technique has been widely used to determine the local atomic vibrational properties of semiconductors.^[^ ^45^ ^]^ Although in an ideal rock‐salt structure of PbTe, none of phonons is first‐order Raman active, the existence of imperfections including grain boundaries could induce local lattice distortions deviating from its ideal NaCl structure thus make the optical phonon modes^[^ ^46^ ^]^ at the Brillouin zone center (Γ) Raman‐active^[^ ^47^ ^]^ (Figure 4a), which has been observed in literature works on polycrystalline PbTe.^[^ ^48^ ^]^ Raman peaks at about 47, 58, 108, and 150 cm^−1^, respectively, correspond to the transverse optical (TO) modes, Eu impurity, longitudinal optical (LO) modes, and TeO_2_.^[^ ^49^ ^]^ The solid curves show the deconvolution according to a Lorentzian approximation.

The broadening in Raman peaks of both TO and LO modes at Γ point enables a direct indication of the broadening in phonon dispersion due to lattice strain fluctuations.^[^ ^49a^ ^]^ Due to the existence of dislocation‐induced lattice strain fluctuations, Na_0.03_Eu_0.03_Cd_0.03_Pb_0.91_Te is found to have a significant frequency broadening in both TO and LO modes as compared to that of Eu_0.03_Cd_0.03_Pb_0.94_Te (Figure 4b). Similar Raman peak broadening due to the existence of lattice strains is frequently observed in many semiconductors.^[^ ^45^ ^]^

Alloying with EuTe and CdTe has been proven to effectively converge the valence bands and to increase the band gap of PbTe. Previous works revealed that the thermoelectric performance is optimized at an alloying concentrate of ≈3%.^[^ ^34^, ^35^ ^]^ This leads the current work to focus on the PbTe‐3%EuTe alloys as the parent material with a further CdTe‐alloying, where Na‐doping is involved in optimizing the carrier concentration and defect structure as detailed above.

**Figure 5:**
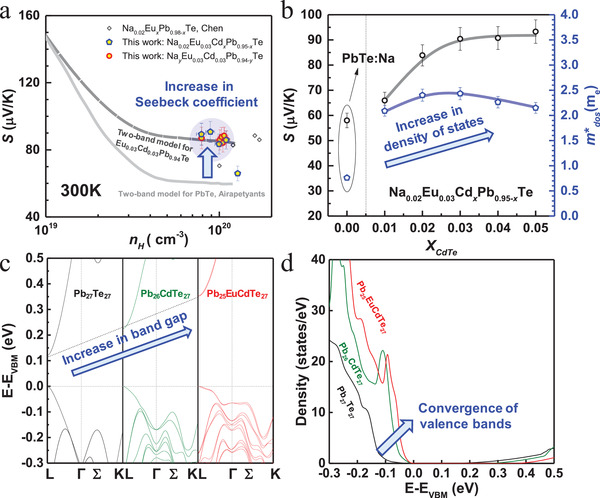
a) Room temperature Seebeck coefficient (*S*) versus Hall carrier concentration (*n_H_*) for various PbTe alloys from this work and literatures, with a comparison to predictions according to the two‐valance‐band model; b) Seebeck coefficient and density of states mass at room temperature for Na_0.02_Eu_0.03_Cd*_x_*Pb_0.95−_ *_x_*Te; c) calculated band structures and d) density of states for Pb_27_Te_27_, Pb_26_CdTe_27_, and Pb_25_EuCdTe_27_ with a setting of valence band maximum (VBM) at 0 eV.

**Figure** **5**a shows the room temperature carrier concentration dependent Seebeck coefficient for various series of PbTe alloys from this work and the literatures.^[^ ^16^, ^35^ ^]^ It is shown that, alloying with EuTe and CdTe in this work indeed leads to an effective increase in Seebeck coefficient and band gap (Figure 5; Figure S7a, Supporting Information). A two‐band model with a reduced band offset of 0.09 eV is used to estimate the relationship between Seebeck coefficient and carrier concentration for Eu_0.03_Cd_0.03_Pb_0.94_Te alloys (Figure 5a), which shows a good agreement with the measurements. This could be understood by the CdTe‐ and EuTe‐alloying induced convergence of *L* and Σ valence bands, as evidenced from the increase of the ratio of density‐of‐states mass (m*_DOS_) to Durde mass (m*_Drude_) (Figure 5b; Figure S8, Supporting Information) and band structure calculations on a supercell of 54 atoms (Figure 5c). The details on density functional theory (DFT) calculations are given in the Supporting Information. It is seen that the valence band maximum (VBM) and the conduction band minimum (CBM) locate at *L* point for Pb_27_Te_27_, Pb_26_CdTe_27_, and Pb_25_EuCdTe_27_. In addition, the direct band gap at *L* increases due to CdTe‐ and EuTe‐alloying, which is consistent with the optical measurements (Figure S7a, Supporting Information). Importantly, alloying effectively reduces the energy offset between the *L* and Σ valence bands, leading to an increase in density of states (DOS) near the valence band edge (Figure 5d) thus an effective involvement of both bands for charge transport. This results in a superior electronic performance as observed in the literatures.^[^ ^18^, ^24^, ^27^ ^]^ Note that m*_Drude_ (Table S2, Supporting Information) estimated by optical measurements (Figure S8e,f, Supporting Information) remains nearly unchanged upon Na‐doping induced dislocations (Figures 2 and 3a), further indicating a nearly pure thermal effect of the dislocations induced lattice strain fluctuations in this work (Figure 3d).

**Figure 6:**
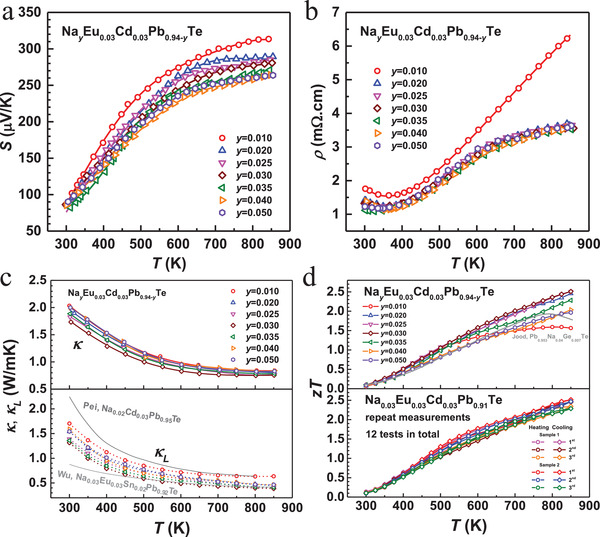
a) Temperature‐dependent Seebeck coefficient, b) resistivity, c) total and lattice thermal conductivity and d) figure of merit for Na*_y_*Eu_0.03_Cd_0.03_Pb_0.94−_ *_y_*Te, with a comparison to literature results.^[^ ^13^, ^31^, ^34^ ^]^

Temperature‐dependent Hall coefficient and Hall mobility for Na_0.02_Eu_0.03_Cd*_x_*Pb_0.95−_ *_x_*Te (*x* ≤ 0.05) and Na*_y_*Eu_0.03_Cd_0.03_Pb_0.94−_ *_y_*Te (*y* ≤ 0.05) are shown in Figure S5, Supporting Information, and the Hall mobility measurements suggest an unchanged dominant carrier scattering by acoustic phonons. Note that the carrier mobility is nearly unaffected by dislocations (lattice strains) in the temperature range studied in this work, which is presumably due to the high dielectric constant of PbTe for a strong screening effect of Coulombic scattering by charged dislocation.^[^ ^50^ ^]^ Temperature‐dependent Seebeck coefficient, resistivity, total, and lattice thermal conductivity (*κ_L_*) and thermoelectric figure of merit (*zT*) for Na*_y_*Eu_0.03_Cd_0.03_Pb_0.94−_ *_y_*Te (*y* ≤ 0.05) are shown in **Figure** **6**.

The property hysteresis between heating and cooling profiles (Figure S9, Supporting Information) in the temperature range of 500–700 K is mainly due to the solubility change of CdTe in PbTe resulting a transition between precipitation and redissolution. Since CdTe‐alloying leads to a strong valence band movement, a hysteresis on *S* and ρ can be observed. Similar effects are also found in PbTe‐CdTe^[^ ^34^ ^]^ and SnTe‐CdSe.^[^ ^24a^ ^]^ Importantly, the existence of temperature‐induced solubility change here does not lead to an obvious change in *zT* (Figure 6d). Seebeck coefficient and resistivity for the high‐*zT* sample Na_0.03_Eu_0.03_Cd_0.03_Pb_0.91_Te are consecutively measured at 850 K for 1440 min (Figure S10, Supporting Information). A slight change of <3% on both *S* and ρ nicely suggests the stability of the thermoelectric performance at this high temperature. Moreover, the transport properties for Na_0.03_Eu_0.03_Cd_0.03_Pb_0.91_Te annealed for different durations (up to two months), are measured and shown in Figure S11, Supporting Information. The highly comparable transport properties confirm the reproducibility, which suggests the good stability.

As compared to the literature results for Na_0.02_Cd_0.03_Pb_0.95_Te,^[^ ^34^ ^]^ the reduction in *κ_L_* observed in this work (Figure 6c) could be understood by the additional phonon scattering by dislocations. However, *κ_L_* obtained in this work at *T* < 500 K is higher than that of Na_0.03_Eu_0.03_Sn_0.02_Pb_0.92_Te,^[^ ^13a^ ^]^ which is believed to be the result of the formation of CdTe precipitates rather than a solid solution at these temperatures. Both effects of superior electronic performance guaranteed by converged valence bands and low lattice thermal conductivity enabled by dislocation‐induced lattice strains (Figure S7b, Supporting Information) successfully lead to a realization of an extraordinary and highly reproducible *zT* (Figure 6; Figure S11, Supporting Information). The high *zT* obtained in this work, particularly at *T* < 673 K (safely measurable temperature range of the module made here), is further demonstrated by a 2 × 4 pair thermoelectric module using p‐Na_0.03_Eu_0.03_Cd_0.03_Pb_0.91_Te and *n*‐Yb_0.3_Co_4_Sb_12_ showing both comparable conversion efficiency and output power under temperature gradients of 270 and 370 K to the literature results^[^ ^31b^ ^]^ (Figure S12, Supporting Information) having comparable *zT* (Figure 6d).^[^ ^31b^ ^]^

## 3 Summary

In this work, manipulation of both doping and alloying regulates the defect and band structure of PbTe thermoelectrics for a synergistic improvement in electronic and phononic properties, leading to a successful revelation of an extraordinary thermoelectric figure of merit. Electronically, it is illustrated here that monontelluride‐alloying offers great capability for converging the valence bands. Thermally, this work demonstrates dense in‐grain dislocations are particularly effective for minimizing the lattice thermal conductivity. In addition, defect induced fluctuations in lattice strain are unveiled to broaden the phonon dispersion for shortening the phonon relaxation time, which might offer an insightful guidance for advancing thermoelectrics.

## Conflict of Interest

The authors declare no conflict of interest.

## Supporting information

Supporting Information
