# Supplementary material for: Thermoelectric Enhancements in PbTe Alloys Due to Dislocation‐Induced Strains and Converged Bands
Source: Adv Sci (Weinh). 2020 May 15;7(12):1902628. doi: 10.1002/advs.201902628 (PMC7312309; doi:10.1002/advs.201902628)
Supplement: Supplementary file 1 — Supporting Information [file ADVS-7-1902628-s001.pdf]

Copyright WILEY-VCH Verlag GmbH & Co. KGaA, 69469 Weinheim, Germany, 2020.

Supporting Information

for *Adv. Sci.,* DOI: 10.1002/advs.201902628

Thermoelectric Enhancements in PbTe Alloys Due to Dislocation-Induced Strains and Converged Bands

Yixuan Wu, Pengfei Nan, Zhiwei Chen, Zezhu Zeng, Ruiheng Liu, Hongliang Dong, Li Xie, Youwei Xiao, Zhiqiang Chen, Hongkai Gu, Wen Li, Yue Chen, Binghui Ge, and Yanzhong Pei**

Supplementary

Thermoelectric Enhancements in PbTe Alloys due to Dislocation-induced Strains and Converged Bands

Yixuan Wu[1,#], Pengfei Nan[2,#], Zhiwei Chen[1], Zezhu Zeng[3], Ruiheng Liu[4], Hongliang Dong[5], Li Xie[4], Youwei Xiao[1], Zhiqiang Chen[5], Hongkai Gu[5,6], Wen Li[1], Yue Chen[3], Binghui Ge[2,*] and Yanzhong Pei[1,*]

[1]Interdisciplinary Materials Research Center, School of Materials Science and Engineering, Tongji Univ., 4800 Caoan Rd., Shanghai 201804, China.

[2]Institute of Physical Science and Information Technology, Anhui University, Hefei 230601, China

[3]Department of Mechanical Engineering, The University of Hong Kong, Pokfulam Road, Hong Kong SAR, China

[4]State Key Laboratory of High Performance Ceramics and Superfine Microstructure, Shanghai Institute of Ceramics, CAS, Shanghai 200050, China

[5]Center for High Pressure Science & Technology Advanced Research, Shanghai 201203, China

[6]State Key Laboratory of Superhard Materials, Jilin University, Changchun 130012, China

[#]These authors contribute equally to this work

[*]Email: bhge@ahu.edu.cn (BG); yanzhong@tongji.edu.cn (YP)

Materials and Methods:

Polycrystalline PbTe, $Cd_{0.03}Eu_{0.03}Pb_{0.94}Te$, $Na_{0.02}Eu_{0.03}Cd_xPb_{0.95-x}Te$ (x≤0.05) and $Na_yEu_{0.03}Cd_{0.03}Pb_{0.94-y}Te$ (y≤0.05) were synthesized by melting stoichiometric high-purity elements Pb, Eu, Cd, Na and Te (>99.99%) at 1300 K for 3 hours and quenching in cold water, then annealing at 900 K. In order to check the dislocation stability, the high performance sample was annealed for longer up to 2 months. The annealed ingots were hand ground into powders for hot pressing and identifying the phase compositions. The powders were densified by hot pressing under a uniaxial pressure of ~60 MPa at 877 K for 30 minutes. The obtained pellets were ~12 mm in diameter and ~1.6 mm in thickness for property measurements.

X-ray diffraction (Synchrotron XRD, λ=0.6888 Å and laboratory XRD, DX2700, Cu-$K\alpha$, λ=1.5406 Å) were used to identify the phase compositions and to estimate the lattice strain. Synchrotron XRD measurements were carried out with the beamline No. 14B of the Shanghai Synchrotron Radiation Facility. The energy of the monochromatic X-ray beam was 18 keV. Optical reflectance was characterized by a Fourier Transform Infrared Spectroscopy (FTIR, Bruker Tensor II equipped with a diffuse reflectance attachment) at room temperature. Bright Field (BF) in the Scanning Transmission Electron Microscopy (STEM) mode were used to characterize the microstructure. STEM specimens were prepared by traditional mechanical polishing, dimpling and ion milling using liquid nitrogen. The Raman spectra were excited by the 532 nm lines of an argon laser in the back-scattering geometry, using a Jobin Yvon model U-1000 monochromator equipped with a conventional photo-counting system. The sintered pellet samples were held onto a glass slide for Raman measurements. In order to ensure a better signal-to-noise ratio, 3 sets of measurements were counted for averaging.

The thermal conductivity (κ) was calculated by $\kappa = \lambda C_p d$; where λ was the thermal diffusivity measured by a laser flash technique (Netzsch LFA457), d was the density, C_p was determined by the measured values of Blachnik and Igel $C_p(k_B/\text{atom})=3.07+0.00047(T/\text{K}-300)$ for lead chalcogenides[1]. The electronic transport properties including Seebeck coefficient (S), Hall coefficient (R_H), and resistivity ($\rho=1/\sigma$) were simultaneously measured under helium. The Seebeck coefficient was obtained from the slope of the voltage versus temperature gradients of 3-5 K; the resistivity and Hall coefficient were measured using the van der Pauw technique with a reversible magnetic field of 1.5 T. The measurement uncertainty of S, ρ and κ is estimated to be about 5%.

Density functional theory (DFT) calculations were performed with VASP [2] using Perdew-Burke-Ernzerhof functional (PBE) of projector augmented wave (PAW) method[3]. A 3×3×3 supercell of the rock-salt primitive cell was used to study the effects of alloying on the band structure and density of states of PbTe. For $Pb_{26}CdTe_{27}$, one Pb atom in the supercell was substituted by the alloying atom arbitrarily, whereas quasi-random structures were generated for $Pb_{25}EuCdTe_{27}$ using USPEX[4]. An energy cutoff of 400 eV was applied. We used the Γ-centered Monkhorst-Pack k-point meshes [5] of 4 × 4 × 4 for self-consistent calculations and 5 × 5 × 5 for DOS calculations. Spin-orbit coupling (SOC) was considered for all systems. The convergence criterion was set to 10^{-6} eV for band structure and DOS calculations.

Mechanical strength was measured by a modified small punch (MSP) technique (AGX-X-10kN). The MSP strength σ_{MSP} was estimated by: $\sigma_{MSP} = 3P_{max}/(2\pi t^2)[1-(1-r^2)/4\times b^2/a^2+(1+r)\ln(a/b)]$, where P_{max} is the maximum load prior to failure, t is the thickness of the sample, r is the Poisson's ratio, a is radius of the center hole and b is the radius of pressure head, respectively. The Vickers hardness (H_V) were determined by Vickers indentation technique (FV-700, Future-Tech Corporation) at a load of 0.3 Kgf (2.94 N) with a dwell of 5 seconds on polished surfaces. The hardness (H_V) was estimated from the diagonal length of the indentation using Equation: $H_V=1.854W/d^2$, where W is the applied load and d is the mean value of the diagonal length[6]. Vickers hardness and MSP strength were obtained by averaging 5 measurements.

Module fabrication and measurements

A 2×4 pair module using p-$Na_{0.03}Eu_{0.03}Cd_{0.03}Pb_{0.91}Te$ in this work and n-$Yb_{0.3}Co_4Sb_{12}$ was assembled by soldering. Both p- and n-type legs were diced into desired dimensions (4×4×8 mm^3 for p-type and 2×2×8 mm^3 for n-type) and bridged by a copper-clad plate (CCL) at the hot side. The cold side was welded to a direct bonding copper (DBC) substrate using a tin-based solder. A layer of nickel ~5 μm exists as a diffusion barrier layer between the electrode and thermoelectric materials. The overall dimension of the module is about 20×20×10 mm^3. The power output and the conversion efficiency were measured using a home-built system under Ar atmosphere. The hot side of the module was heated up to 673 K and the cold side was stabilized at 303 K. The electrical power output and conversion efficiency of the module were measured at hot-side temperatures of 523 K, 573 K, 623 K, and 673 K, respectively. The conversion efficiency is estimated by:

$$\eta = \frac{P}{P+Q_c}\times 100\% \qquad \text{Equation (S1)}$$

where P is the electrical output power of the module and Q_c is the heat flow measured by a flux probe at the cold end.

Estimation of effective mass

1. Conductivity mass (m^*_{Drude}):

Once electrical conductivity and carrier concentration are known, the conductivity effective mass can be approximately determined from plasma frequency (ω_p)[7]:

$$\omega_p^2 = \frac{ne^2}{\epsilon_\infty m^*_{Drude}} \qquad \text{Equation (S2)}$$

where ϵ_∞=33 ϵ_0 is the dielectric constant of the high-frequency limit, e is the elementary charge, n is the free-carrier concentration.

2. Density-of-states mass (m^*_{Dos}):

The simplest description of Seebeck coefficient of metals and degenerate semiconductors by density-of-states mass (m^*_{Dos}) can be approximated by [7b]:

$$S = \frac{8\pi^2 k_B^2}{3e\,h^2} m^*_{Dos} T \left(\frac{\pi}{3n}\right)^{\frac{2}{3}} \qquad \text{Equation (S3)}$$

where n is the carrier concentration, T is the temperature, e is the elementary charge, k_B is the Boltzmann constant and h is the Planck constant.

Lattice thermal conductivity model:

Considering the intrinsic lattice anharmonicity and extrinsic mass and strain fluctuations, the lattice thermal conductivity (κ_L) and the relaxation time (τ) are determined by: [8].

$$\kappa_L = \frac{1}{3}\int_0^{\omega_a} C_V(\omega) v_g^2 \tau d\omega \qquad \text{Equation (S4)}$$

$$\tau^{-1} = \tau_\varepsilon^{-1} + \tau_M^{-1} = \gamma^2\varepsilon^2 + \left(\frac{1}{\bar{M}}\right)^2 \Delta M^2 \qquad \text{Equation (S5)}$$

where C_V is the specific heat, ω_a is the cut-off frequency of acoustic phonons, v_g is the phonon group velocity and $\bar{M}$ is the average atomic mass. ε and ΔM respectively represent the strain and mass fluctuations. This model takes only into account the contribution of acoustic phonons, because of their dominant contribution to κ_L [9]. The average Grüneisen parameter is estimated according to the Leont'ev method[10].

The total lattice strains (ε) and mass fluctuations (ΔM) are given by [11]:

$$\varepsilon^2 = A + B\varepsilon_{PD}^2 + C\varepsilon_{DS}^2 \qquad \text{Equation (S6)}$$

$$\Delta M^2 = D(\Delta M_{PD})^2 + E(\Delta M_{DS})^2 \qquad \text{Equation (S7)}$$

where A (for Umklapp scattering), B, C, D and E are the collection of physical constants, and subscripts PD for point defects and DS for dislocations. The pre-factor A is given by:

$$A = \frac{2}{(6\pi^2)^{\frac{1}{3}}} \frac{k_B \bar{V}^{\frac{1}{3}} \omega^2 T}{\bar{M} v_g v_p^2} \qquad \text{Equation (S8)}$$

where k_B is the Boltzmann constant, $\bar{V}$ is the average atomic volume, ω is the phonon frequency, T is the absolute temperature, v_p is the phase velocity, respectively. For simplicity, a Debye dispersion is used in this work.

According to Klemens[12], the pre-factor B due to point defects is:

$$B = \frac{\bar{V}\omega^4}{4\pi v_g v_p^2} 8(1+Q)^2 \qquad \text{Equation (S9)}$$

where Q=3.2 for an exclusion of the anharmonicity of the nearest elastic constants.

The strain field due to point defects can be determined by X-ray diffraction measurements (shift of diffraction peaks) [12]:

$$\varepsilon_{PD}^2 = \sum_i x_i \left(\frac{R_i - \bar{R}}{\bar{R}}\right)^2 = \sum_i x_i \left(\frac{11+r}{31-r}\right)^2 \left(\frac{a_i - \bar{a}}{\bar{a}}\right)^2 \qquad \text{Equation (S10)}$$

where x_i is the concentration of the solute i, R_i is the ionic radii of solute i, $\bar{R}$ is the average ionic radii, r is the Poisson ratio (estimated based on the Leont'ev method[10]), a_i is the lattice constant of solute i and $\bar{a}$ is the average lattice constant, respectively.

Utilizing a Gaussian approximation, XRD rocking curves enable an estimation on lattice strain fluctuations. The full width at half maximum of the broadened XRD peaks due to lattice strains (β_ε) is given by[13]:

$$\beta_\varepsilon^2 = 8\left(\overline{\varepsilon_{DS}^2}\right)\ln 2\,(\tan\theta)^2 \qquad \text{Equation (S11)}$$

where θ is the Bragg angle. The mean square strain ($\overline{\varepsilon_{DS}}^2$) along the direction $\bar{n}$ in the redial plane of the dislocation ($\bar{n}$ is the radial component of $\bar{N}$) is:

$$\overline{\varepsilon_{DS,n}^2} = \frac{1}{\pi l^2}\int_0^{2\pi}\int_{l_0}^{l} l\,[\varepsilon_{ll} + \varepsilon_{l\theta}\sin(\delta-\theta)\cos(\delta-\theta)]^2 dl d\theta = \frac{3-2r+3r^2+2(r^2-1)\cos(2\delta)}{32\pi^2(r^2-1)^2}\frac{b^2}{l^2}\ln\frac{l}{l_0} \qquad \text{Equation (S12)}$$

Where b is the Burgers vector, l and l_0 are the upper and lower integration limits of the strain field, ε_{ll}=$b\sin(\theta)/4\pi(1+r)l$ and $\varepsilon_{l\theta}$

Supplementary

$=b\cos(\theta)/2\pi(1-r)l$. l and l_0 are typically defined as $1/2N_D^{1/2}$ and one Burgers vector. Defining Δ as the angle between the dislocation glide plane normal and $\bar{N}$, and φ as the angle between $\bar{N}$ and $\bar{b}$, then $\cos^2(\delta)=\cos^2(\varphi)/(\cos^2(\varphi)+\cos^2(\Delta))$ where the angular relationships are shown in the literature[13]. The mean square strain along $\bar{N}$ is then $\overline{\varepsilon_{DS,N}}^2=\overline{\varepsilon_{DS,n}}^2(\cos^2(\varphi)+\cos^2(\Delta))$. The random orientation of dislocations is confirmed by our STEM observations, which rationalizes the following mathematical average as: both the integral average value of $\cos^2(\delta)$, $\cos^2(\varphi)$ and $\cos^2(\Delta)$ are 1/2. This leads $\cos(2\delta)$ to be zero.

This work utilizes Carruthers' Equation[14] to estimate the phonon relaxation time by edge dislocation scattering. This leads the pre-factor C to be:

$$C=\frac{2}{3}\frac{32\pi^2(r^2-1)^2}{3-2r+3r^2}\omega\ln\frac{\frac{1}{2N_D^{\frac{1}{2}}}}{b} \qquad \text{Equation (S13)}$$

where N_D is the dislocation density. The dislocation density can be solved when the lattice strain due to dislocations and the Burgers vector are known.

For the mass term due to point defects, the pre-factor D and ΔM_{PD} are[12]:

$$D=\frac{\bar{V}\omega^4}{4\pi v_g v_p^2} \qquad \text{Equation (S14)}$$

$$\Delta M_{PD}^2=\sum_i x_i(M_i-\bar{M})^2 \qquad \text{Equation (S15)}$$

And the mass term for dislocations, the pre-factor E is [15]:

$$E=\frac{\bar{V}^{\frac{4}{3}}\omega^3}{v_g v_p} \qquad \text{Equation (S16)}$$

$$\Delta M_{DS}^2=N_D(0-\bar{M})^2 \qquad \text{Equation (S17)}$$

Parameters used for the modeling are listed in Table S1.

Table S1. Parameters used for the modeling.

Parameters	Description	Quantity	Ref.
a_i	Lattice parameters for solute i	a_{EuTe}=6.59 Å	[16]
a	Average lattice parameters for the alloy	From Figure S1	This work
Npri	Number of atoms in primitive cell	2	-
$\bar{V}$	Average atomic volume of the alloy	$\bar{a}^3/8$ m^3	-
M	Average atomic mass of the alloy	$M_{EuxPb1\text{-}x\text{Te}}/(N\text{pri}\times6.023\times10^{23})$ kg	-
M_i	Average atomic mass of impurities	$M_{EuTe}/(N\text{pri}\times6.023\times10^{23})$ kg	-
θ_D	Debye temperature	120 K	[17]
ω_a	Acoustic cut-off frequency	$(\theta_D/(N\text{pri})^{1/3})/(\hbar/k_B)$ Hz	-
γ	Gruneisen parameter	1.66	This work
x_i<br>z_i	Impurity concentrations	z_{Eu} = 0.03	This work
r	Poisson's ratio	0.281	This work
b	Burgers vector	$a/2$	This work

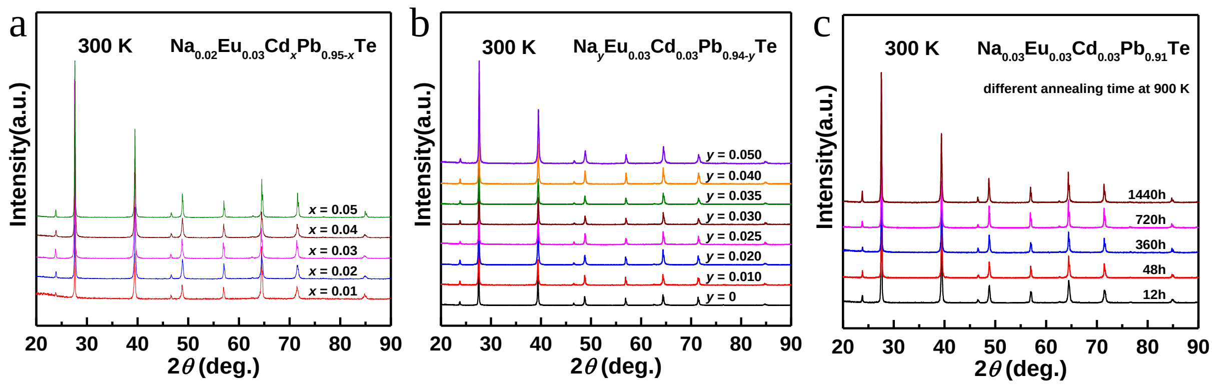

Figure S1. Room-temperature XRD patterns for $Na_{0.02}Eu_{0.03}Cd_xPb_{0.95-x}Te$ (a) and $Na_yEu_{0.03}Cd_{0.03}Pb_{0.94-y}Te$ (b), as well as the high-performance material $Na_{0.03}Eu_{0.03}Cd_{0.03}Pb_{0.91}Te$ annealed at 900 K for different durations (c).

Supplementary

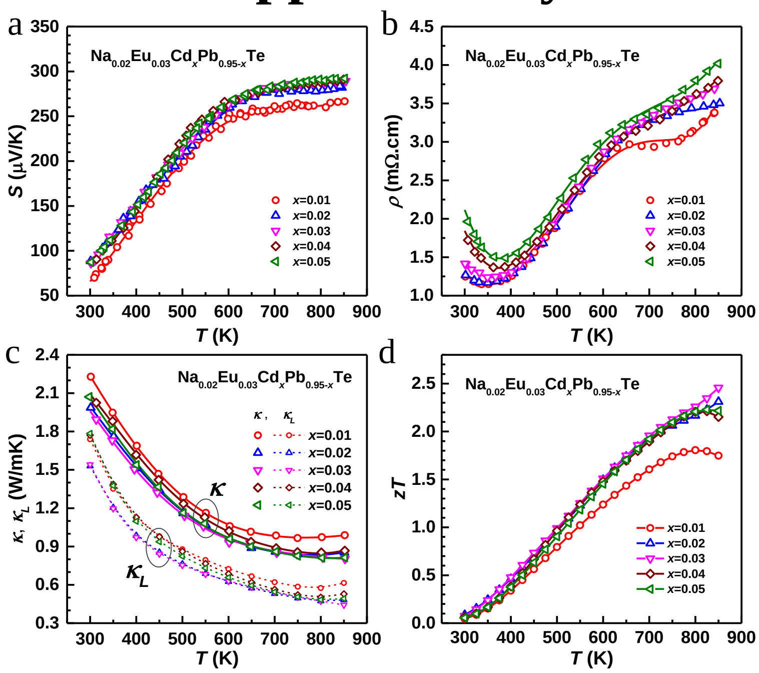

Figure S2. Temperature dependent Seebeck coefficient (a), resistivity (b), total and lattice thermal conductivity (c) and thermoelectric figure of merit (d) for $Na_{0.02}Eu_{0.03}Cd_xPb_{0.95-x}Te$.

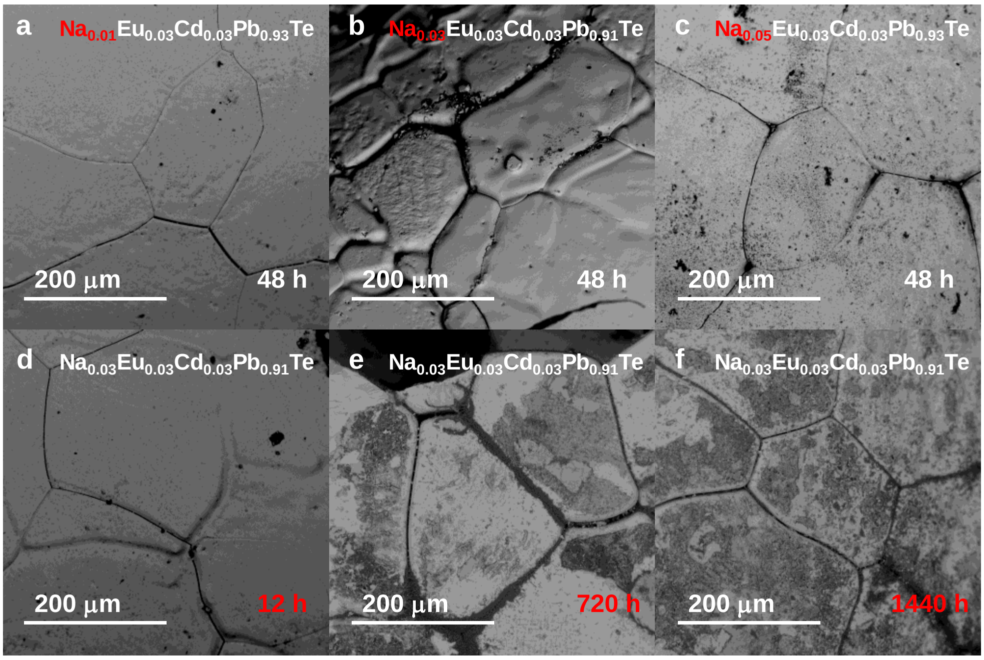

Figure S3. Scanning electron microscopy images for $Na_yEu_{0.03}Cd_{0.03}Pb_{0.94-y}Te$ and $Na_{0.03}Eu_{0.03}Cd_{0.03}Pb_{0.91}Te$ with different annealing times, indicating the comparable grain sizes in all samples.

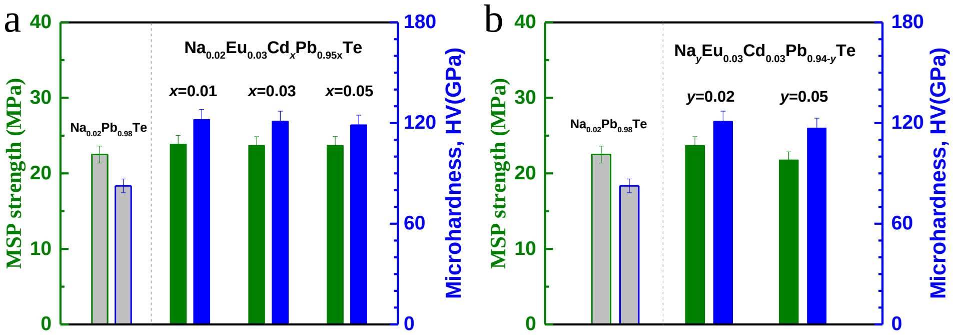

Figure S4. Vickers hardness and strength by a modified small punch (MSP) technique for $Na_yEu_{0.03}Cd_{0.03}Pb_{0.94-y}Te$ and $Na_{0.02}Eu_{0.03}Cd_xPb_{0.95-x}Te$.

Supplementary

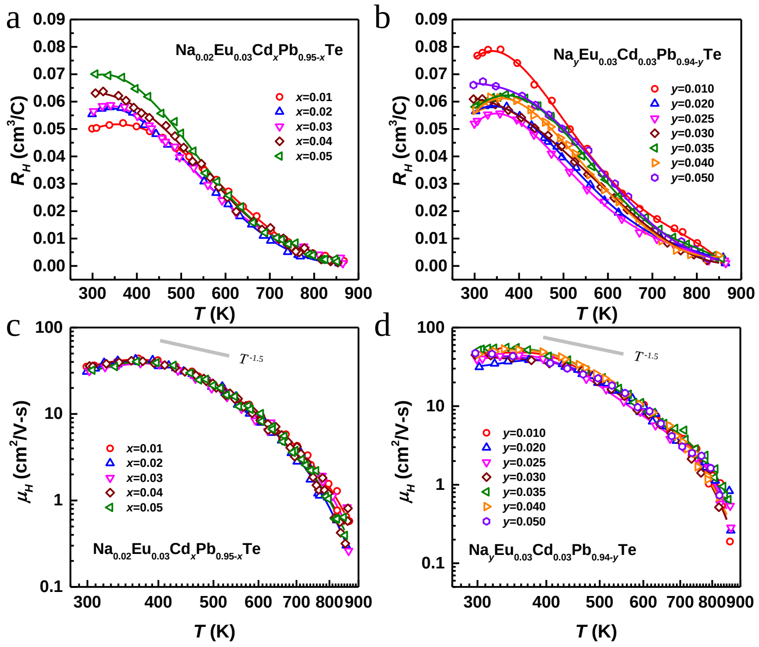

Figure S5. Temperature dependent Hall coefficient (a, b) and Hall mobility (c, d) for $Na_{0.02}Eu_{0.03}Cd_xPb_{0.95-x}Te$ (a, c) and $Na_yEu_{0.03}Cd_{0.03}Pb_{0.94-y}Te$ (b, d).

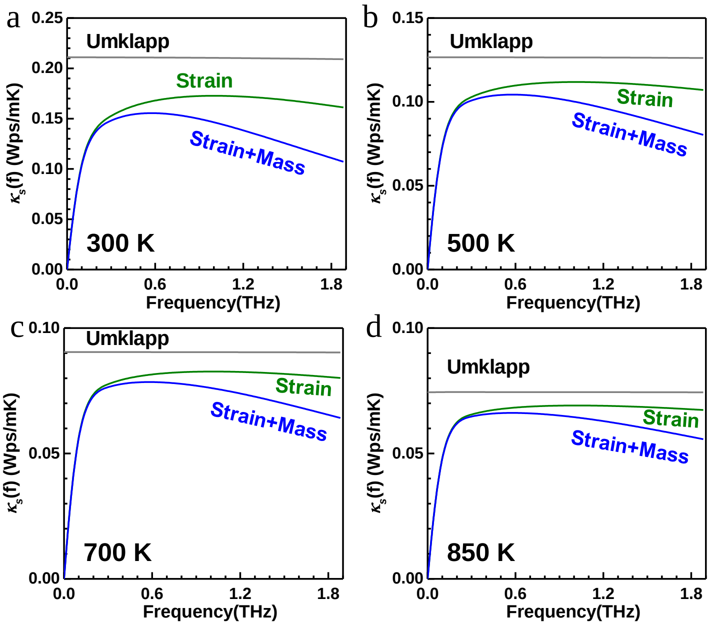

Figure S6. Spectral lattice thermal conductivity at 300 (a), 500 (b), 700 (c) and 850 K (d) for $Na_{0.03}Eu_{0.03}Cd_{0.03}Pb_{0.91}Te$

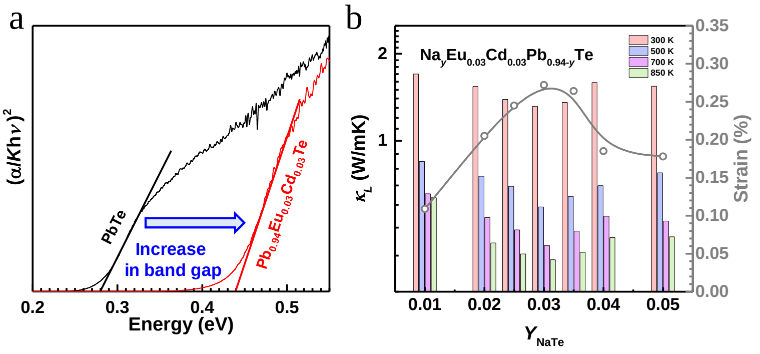

Figure S7. Room temperature optical absorption as a function of photon energy for PbTe and $Eu_{0.03}Cd_{0.03}Pb_{0.91}Te$ (a); annealing time dependent lattice strains and lattice thermal conductivity (κ_L) at different temperatures for $Na_yEu_{0.03}Cd_{0.03}Pb_{0.94-y}Te$.

Supplementary

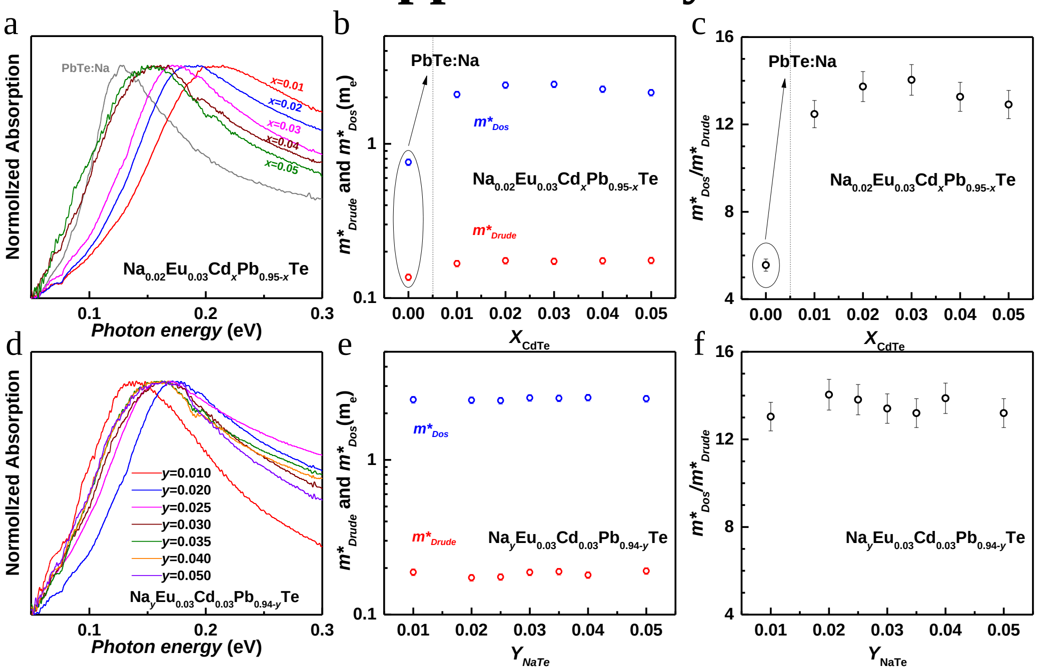

Figure S8. Normalized infrared reflectance versus photon energy and at room temperature for PbTe:Na, $Na_{0.02}Eu_{0.03}Cd_xPb_{0.95-x}Te$ and $Na_yEu_{0.03}Cd_{0.03}Pb_{0.94-y}Te$ (a, d); The Drude effective mass (m^*_{Drude}) estimated by IR measurements and density-of-states effective mass (m^*_{DOS}) determined according to Equation S3 (b, e) and the ratio between (c, f).

Table S2. Room temperature Hall carrier concentration (n_H), conductivity effective mass (m^*_{Drude}), plasma energy and density (g/cm^3) for $Na_{0.02}Eu_{0.03}Cd_xPb_{0.95-x}Te$ and $Na_yEu_{0.03}Cd_{0.03}Pb_{0.94-y}Te$.

Materials	n_H (10^{20}cm^{-3})	m^*_{Drude} (m_e)	Plasma energy (eV)	Density (g/cm^3)
PbTe:Na	0.36	0.13	0.13	8.21
$Na_{0.02}Eu_{0.03}Cd_{0.01}Pb_{0.94}Te$	1.28	0.16	0.20	8.11
$Na_{0.02}Eu_{0.03}Cd_{0.02}Pb_{0.93}Te$	1.10	0.17	0.18	8.08
$Na_{0.02}Eu_{0.03}Cd_{0.03}Pb_{0.92}Te$	0.99	0.17	0.17	8.03
$Na_{0.02}Eu_{0.03}Cd_{0.04}Pb_{0.91}Te$	0.89	0.17	0.16	8.07
$Na_{0.02}Eu_{0.03}Cd_{0.05}Pb_{0.90}Te$	0.79	0.17	0.15	8.06
$Na_{0.01}Eu_{0.03}Cd_{0.03}Pb_{0.93}Te$	0.79	0.19	0.14	8.11
$Na_{0.025}Eu_{0.03}Cd_{0.03}Pb_{0.915}Te$	1.01	0.17	0.17	8.06
$Na_{0.03}Eu_{0.03}Cd_{0.03}Pb_{0.91}Te$	1.03	0.17	0.16	8.03
$Na_{0.035}Eu_{0.03}Cd_{0.03}Pb_{0.905}Te$	1.05	0.18	0.16	8.00
$Na_{0.04}Eu_{0.03}Cd_{0.03}Pb_{0.90}Te$	1.07	0.18	0.17	8.06
$Na_{0.05}Eu_{0.03}Cd_{0.03}Pb_{0.89}Te$	1.07	0.18	0.16	8.07

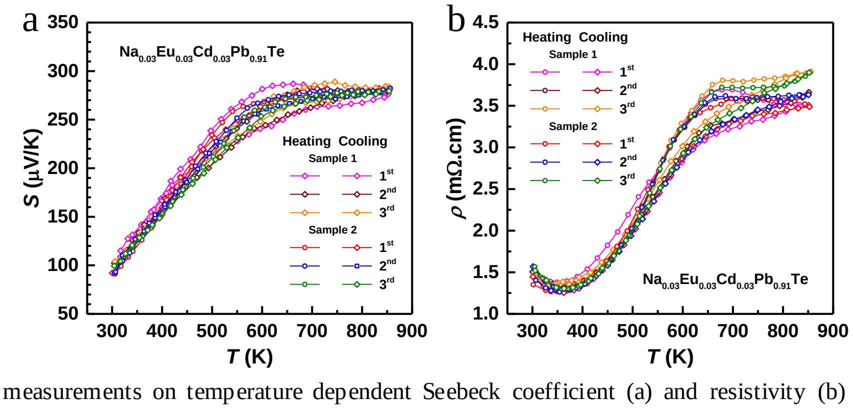

Figure S9. Repeated measurements on temperature dependent Seebeck coefficient (a) and resistivity (b) for the high-zT composition $Na_{0.03}Eu_{0.03}Cd_{0.03}Pb_{0.91}Te$. Note here that the property hysteresis between heating and cooling profiles are mainly due to the highly temperature dependent solubility of CdTe in PbTe.

Supplementary

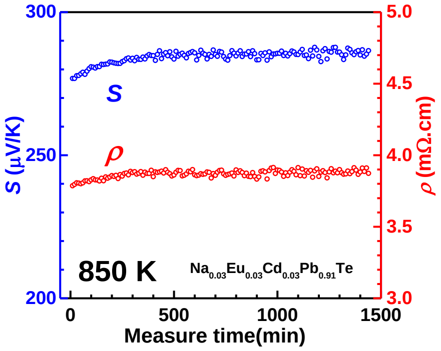

Figure S10. Time-dependent Seebeck coefficient and resistivity for $Na_{0.03}Eu_{0.03}Cd_{0.03}Pb_{0.91}Te$ at 850 K.

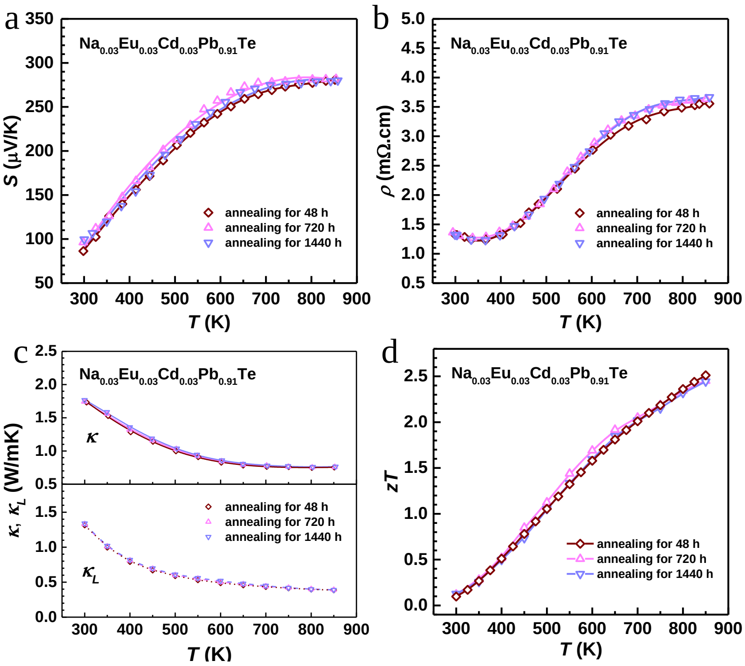

Figure S11. Temperature dependent Seebeck coefficient (a), resistivity (b), total and lattice thermal conductivity (c) and figure of merit (d) for the high-zT material $Na_{0.03}Eu_{0.03}Cd_{0.03}Pb_{0.91}Te$ annealed for different durations.

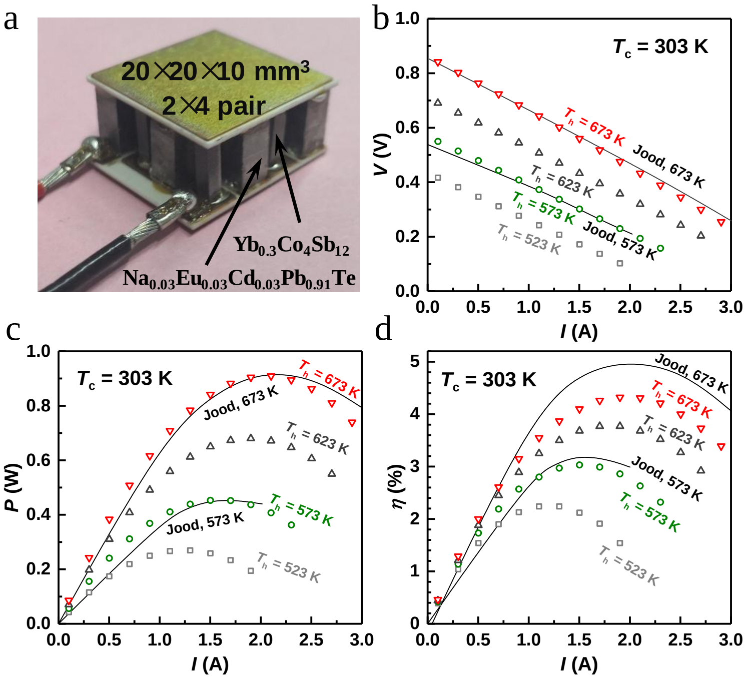

Figure S12. A photo of a 2×4 pair thermoelectric module using p-$Na_{0.03}Eu_{0.03}Cd_{0.03}Pb_{0.91}Te$ here and n-$Yb_{0.3}Co_4Sb_{12}$ (a) and the corresponding output I-V curve (b), electrical power output (c) and the conversion efficiency (d) under temperature gradients of 220, 270, 320 and 370 K, with a comparison to the literature results[18].

Supplementary

Supplementary References:

[1] a) Y. Pei, A. D. LaLonde, H. Wang, G. J. Snyder, *Energy Environ. Sci.* **2012**, 5, 7963; b) R. Blachnik, R. Igel, *Z Naturforsch B* **1974**, 29, 625.

[2] a) G. Kresse, J. Hafner, *Phys. Rev. B.* **1993**, 47, 558; b) G. Kresse, J. Furthmüller, *Phys. Rev. B.* **1996**, 54, 11169.

[3] P. E. Blöchl, *Phys. Rev. B.* **1994**, 50, 17953.

[4] A. R. Oganov, C. W. Glass, *J Chem Phys* **2006**, 124, 244704.

[5] H. J. Monkhorst, J. D. Pack, *Phys. Rev. B.* **1976**, 13, 5188.

[6] X. Liu, Y.-C. Fan, J.-L. Li, L.-J. Wang, W. Jiang, *Adv Eng Mater* **2015**, 17, 28.

[7] a) I. Hamberg, C. G. Granqvist, *J. Appl. Phys.* **1986**, 60, R123; b) M. Cagnoni, D. Führen, M. Wuttig, *Adv. Mater.* **2018**, 30, 1801787.

[8] P. G. Klemens, **1958**, 7, 1.

[9] E. S. Toberer, A. Zevalkink, G. J. Snyder, *J. Mater. Chem.* **2011**, 21, 15843.

[10] K. KEONTEV, *Sov Phys Acoust+* **1981**, 27, 309.

[11] Y. Wu, Z. Chen, P. Nan, F. Xiong, S. Lin, X. Zhang, Y. Chen, L. Chen, B. Ge, Y. Pei, *Joule* **2019**, 3, 1276.

[12] P. G. Klemens, *Proceedings of the Physical Society* **1955**, A68, 1113.

[13] M. Hordon, B. Averbach, *Acta Metall Mater* **1961**, 9, 237.

[14] P. Carruthers, *Phys. Rev.* **1959**, 114, 995.

[15] P. G. Klemens, *Solid State Phys.* **1958**, 7, 1.

[16] V. P., SpringerMaterials, 2016.

[17] D. T. Morelli, V. Jovovic, J. P. Heremans, *Phys Rev Lett* **2008**, 101, 035901.

[18] P. Jood, M. Ohta, A. Yamamoto, M. G. Kanatzidis, *Joule* **2018**, 2, 1339.
